# Analysis of Near-Field Characteristics on Improved Structures of Double-Slot Antipodal Vivaldi Antenna

**DOI:** 10.3390/s24154986

**Published:** 2024-08-01

**Authors:** Ha Hoang, Minh-Huy Nguyen, Vinh Pham-Xuan

**Affiliations:** 1 Department of Telecommunications Engineering, Ho Chi Minh City University of Technology—Vietnam National University, Ho Chi Minh City 700000, Vietnam; 2 Dassault Systèmes Deutschland GmbH, 64289 Darmstadt, Germany

**Keywords:** electromagnetic propagation, near-field analysis, near-field characteristic, near-field to far-field transformation, impulse response, structural effect, Vivaldi antenna

## Abstract

A characterization of near-field impulse responses based on electromagnetic (EM) near-field data from an EM solver to explore features of the propagation process on a well-known wideband traveling wave antenna—double-slot Vivaldi antenna—is presented in this article. The intensity, propagating time and partitional response characteristics facilitate interpretation of the propagation process and impacts of the antenna partitions on the process. The EM energy flows guided, reoriented and scattered along a sequence of antennas transmitting and radiating segments were recognized. The geometric features of near-field wavefront surfaces supported evaluation of the EM flow proportions and antenna directivity. Impact of the structural section on radiation was also assessed by the partitional far-field response characteristic in frequency and time domains. Supported by many complementary characteristics in the analyses, inherent features of the propagation process were emphasized and false flags were minimized. By this approach, the simplification for the near-field propagation model contributed to enhancing the insight of near-field propagation processes on the double-slot antipodal Vivaldi antennas and enabled optimizing the antenna structure details.

## 1. Introduction

The first Gibson’s Vivaldi planar antenna [1] introduced in 1979 is a directional antenna with its main EM radiation beam following the antenna’s guiding structure. This results in a high gain over a wide bandwidth. In 1988, Gazit proposed an antipodal Vivaldi structure based on a direct parallel excitation strip line [2] instead of a stub slot-based excitation structure to expand in lower frequency band for the antipodal kind of Vivaldis. Later research has proposed a variety of improved structures to enhance antenna performances [3,4,5,6,7], such as changing the shape of the antennas, adding slots or etched shapes on the wings. Such modifications may aid in enhancing the directivity of the main beam and/or to broaden the radiation bandwidth. These improvements can be achieved by leveraging the resonance phenomenon or higher-order scattering of EM propagation flows. Moreover, integrating dielectric or metal structured parts/components along the Vivaldi slot or at the radiating aperture can effectively trap or couple EM energy from the Vivaldi wings [8,9,10,11]. This strategy enables the redirection of EM flows at the radiating aperture into free space, enhancing the overall performance of the antenna system. The double-slot Vivaldi structures were proposed in [12,13,14,15], where the distribution of the two slots forms a core section between the two lateral Vivaldi wings. The parallelism of the two slots and the EM field guiding of the core facilitate the radiation of EM flows, resulting in flattening wavefronts and consequently better directivity.

Based on conventional characteristics, the impacts of an improved structure on antenna performances can be explored through a simple comparison between overall antenna characteristics with and without the improved structure. The characteristics can be estimated or computed using simulation or measurement. However, creating or optimizing an improved structure needs many deep factors, such as diversity and depth of knowledge about relevant structures and materials; the operating mechanisms; characteristics and equivalent models, in the phase of creation, selection or synthesis of a design process, and sensitivity and effectiveness of optimization algorithms in optimization phase. Each overall antenna characteristic is a superposition of the contributions from all parts of the antenna. It is difficult to interpret the correspondence between a structural detail and a response detail to satisfy requirements for the above deep factors by solely inspecting overall characteristics.

Near-field propagation characteristics analysis can be instrumental in strengthening conventional analysis methods [16,17] as it interprets the impact of locally modified structures such as added slots to near-field responses at their local spaces. Evaluation of antennas can be performed using simulation where an EM solver solves Maxwell’s equations applied to a given geometry. There are different types of EM solver such as time-domain solvers (e.g., Finite-Difference Time-Domain or Finite-Integration Technique) and frequency-domain solvers (e.g., Finite Element Method, Method of Moment). Once an EM solver is provided with a proper model and an appropriate setup, the result of the simulation will accurately describe the response of a given geometry. A wide range of structural sizes and customizability of structure modeling demonstrates generality in applications of EM solvers. Informational wealth in near-field data from EM solvers can resolve the shortages mentioned above. However, the bulk of raw near-field data has been challenging for observations and analyses of near-field features.

This work is a development from previous research in [16,17]. Based on the commercial SIMULIA CST Studio Suite^®^ EM solver [18], the double-slot antipodal Vivaldis with or without improved structures were simulated in the time domain with an impulse excitation to generate near-field data for the next post-processes. Characterization was based on the first-cluster propagation analysis, focusing on the magnitude and time of arrival (ToA) of EM field and Poynting vectors. This facilitated both observations and quantitative analyses of the near-field propagation features from both overall and detail perspectives. The analyses unveiled the concentrations and directions of the EM energy flows in different regions of the antennas. Qualitative and quantitative analyses were conducted on characteristics such as intensity, energy flux, wavefront shape and wavefront flatness in specific regions of interest. The propagation features of the improved structures were measured by comparing the characteristics of the different geometries in the improvement process. These impacts were evaluated, serving as the targets for design optimization. Furthermore, the near-field responses within the partitions of antenna structures were characterized into partitional far-field gains in the frequency domain and partitional far-field impulse responses in the time domain. These near-field and locally reflected characteristics were employed as criteria for the analyses, evaluations and optimizations of three improved versions of double-slot antipodal Vivaldi antennas. These improvements included adjustments such as offsetting the excitation strip line, lengthening the core between Vivaldi double-slots and etching comb slots on lateral wings.

The contributions of this work can be summarized as follows:

- Characterization of near-field propagation using impulse response analysis. New characteristics are proposed, including the first-cluster magnitude and ToA of the Poynting vector, and the flatness of the wavefronts.
- Proposal of the partitional near-field to far-field transformation in time and frequency domains. The transformation is applied to sub-structures of the geometry to analyze the impact of individual sub-structures and local near-field propagation features on the overall performance of the entire geometry.
- Application of all these proposed characteristics and the conventional characteristics to the design of three improved versions of double-slot Vivaldis, including Vivaldi with strip line offset, Vivaldi with adjusted core length and Vivaldi with lateral comb slots. Diversity, complementarity and granularity in the analyzability of the characteristics emphasize the intrinsic nature of the near-field propagation process and reduce false possibilities in the analysis. These contribute to enhance the insight of near-field propagation processes on this kind of traveling wave antenna.

The Section 2 presents definitions of near-field propagation characteristics and partitional near-field to far-field characteristics and a comparison with conventional overall characteristics. Section 3 presents the analyses of near-field characteristics on a structure with excitation strip line offset. The analyses of core length impacts on near-field propagation are presented in Section 4. In Section 5, the effectiveness of the comb slots on the lateral wings is demonstrated. Finally, Section 6 concludes the article.

## 2. Near-Field Propagation Characteristics vs. Conventional Overall Characteristics

The near-field propagation features and local impacts on far-field features were characterized using simulated near-field data extracted from the EM solver. These characteristics can be divided into three groups, including characteristics of EM intensity and energy flow distribution, ToA characteristics and partitional near-field to far-field characteristics.

**Figure 1:**
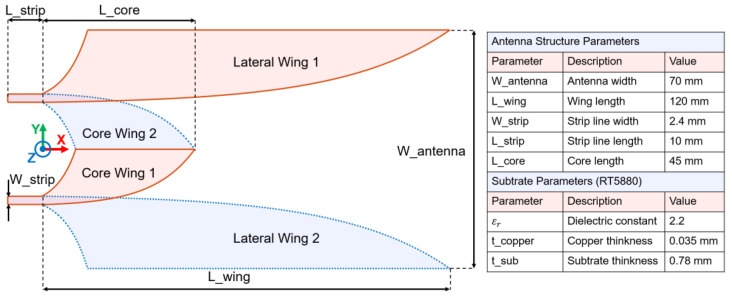
Reference structure and parameters.

**Figure 2:**
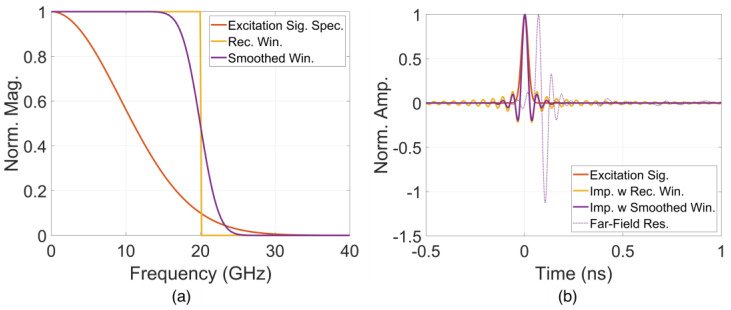
(**a**) Spectrum and (**b**) time signal of excitation, impulses and far-field response.

In this section, the characterization was implemented on a double-slot antipodal Vivaldi antenna modified from [14], as shown in Figure 1. This structure was used as a reference, and its analysis results were compared to those obtained from subsequent analyses of the improved structures. A Gaussian signal with a 0–20 GHz bandwidth was applied simultaneously to two waveguide ports located at the two strip lines’ ends. The signal spectrum of the excitation source signal is shown in Figure 2a,b.

Although the excitation signal exhibits a Gaussian distribution in the frequency spectrum, conventional characteristics such as S11 or gain were assessed using uniform distribution within the 0–20 GHz band. Therefore, to facilitate a comparative analysis of these characteristics, impulse response analyses were also applied within this frequency range. The impulses and responses were processed by the method mentioned in [16,17] using the rectangular and smoothing windows as shown in Figure 2a. The corresponding time impulses and a time response example in far-field are presented in Figure 2b. Due to the prolonged time impulse associated with the rectangular window in the time domain, which complicates observations, the impulse response corresponding to a smooth window was employed instead.

### 2.1. Electromagnetic Intensity and Energy Flow Distribution

**Figure 3:**
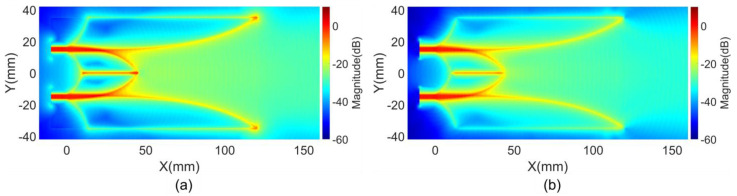
Maximum magnitudes of (**a**) E field and (**b**) H field vectors on reference structure.

The first near-field characteristic, representing the intensity distribution of E and H fields on a selected region of the antenna, was calculated based on the maximum magnitude of E and H field vectors within the analysis space over time during impulse response analysis. The magnitudes of E and H fields were normalized relative to their respective maximum value at the excitation ports. Figure 3 illustrates the maximum magnitude distribution of the E and H field vectors in the middle substrate layer of the reference antenna. As shown in the figure, this characteristic highlights critical regions within the antenna structure where high intensity field flows occur.

**Figure 4:**
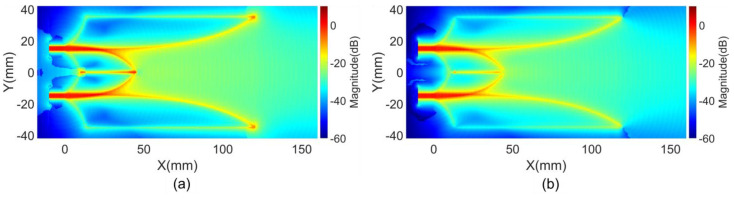
First clusters magnitudes of (**a**) E field and (**b**) H field vectors on reference structure.

The characteristics of the first clusters magnitudes of the E and H field vectors indicate the distribution of these magnitudes within antenna space. These characteristics, presented in Figure 4, show the distribution in the middle substrate layer. Since first clusters typically propagate along the shortest paths, they carry the maximum energy. Consequently, the distribution of the first clusters magnitudes in Figure 4 closely resembles the maximum magnitude distribution shown in Figure 3, except in regions where light-of-sight paths or shortest paths do not support efficient EM transmission. In these areas, the energy of the arriving EM flows from different directions becomes more dominant.

**Figure 5:**
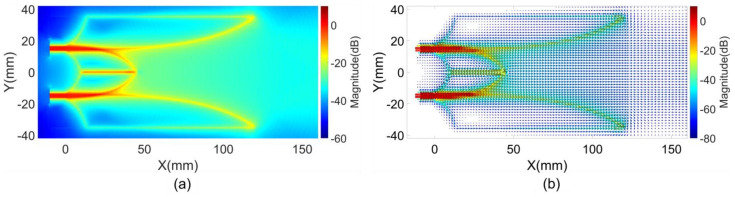
First clusters (**a**) magnitudes and (**b**) directions and magnitudes of Poynting vectors on reference structure.

To supplement the E and H field magnitude characteristics, the magnitude and direction characteristics of the first clusters of Poynting vectors were evaluated and are illustrated in Figure 5. These characteristics demonstrate the distributions of both the strength and direction of energy flows within various regions of the antenna.

The distribution of the magnitude and direction of the E, H fields and Poynting vectors reveals crucial areas within antenna structures that play significant roles in the transmission of EM energy. By observing and conducting enhanced analyses based on these characteristics, it is possible to discern not only EM propagation features such as direction, intensity and the concentration level of EM energy on guiding and orienting structures, but also the impacts of sub-structures of the antenna on EM propagation and scattering.

### 2.2. Time of Arrivals

In impulse response analysis, the ToA of the first clusters is a crucial characteristic for qualitatively analyzing the features of a propagation process. When ToAs at adjacent positions are represented as a contour curve or surface, this delineates a wavefront. Observing contour curves of adjacent ToAs from or around a source allow the identification of energy spreading properties. The impact of objects within the propagation environment, which cause aberrations in energy flows, can also be detected. Characterizing the geometries of these contour curves or surfaces enables the measurement of local or overall propagation features.

**Figure 6:**
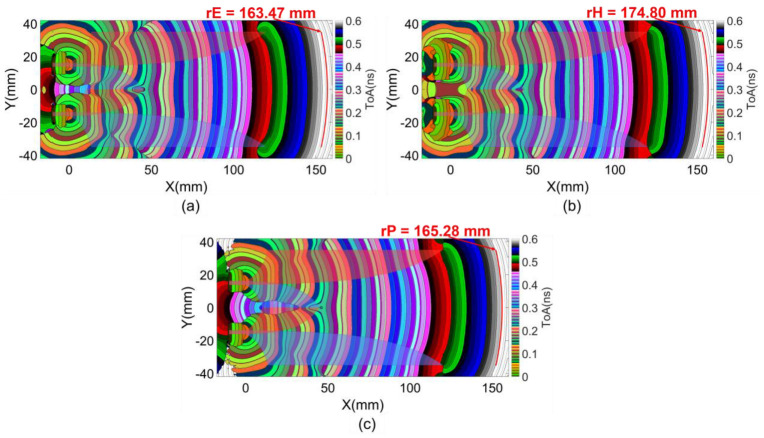
ToAs of first clusters of (**a**) E field, (**b**) H field and (**c**) Poynting vectors on referent structure.

In this work, ToAs were characterized based on the first clusters of E and H field vectors and Poynting vectors. The results at the middle substrate layer of the reference structure are presented in Figure 6. The figure reveals the properties of the energy spreading from the excitation sources and the impacts of the Vivaldi wings and core on the orientation of energy flows. The flatness of the wavefronts can be quantified by the radius of a circle fitted to a wavefront at the radiating antenna aperture, as illustrated in Figure 6. This flatness serves as a near-field characteristic to measure the directivity of the radiating field from the antenna.

### 2.3. Structural Partition for Near-Field to Far-Field Transformation

According to Huygen’s equivalence surface principle, the scattered EM field outside an actual source is equal to an EM field produced by equivalent electric and magnetic current sources on an imaginary surface surrounding the sources [19,20]. It is important to notice that this volume is imaginary and contains only equivalent current sources on its surface, with no fields inside, meaning $E_{I}=0$ and $H_{I}=0$. This principle was applied to transform near-field to far-field based on simulated near-field data extracted on an equivalent surface, denoted as $E_{S}$ and $H_{S}$. The equivalent electric and magnetic currents, $J_{S}$ and $M_{S}$, are required to fulfil the boundary conditions

(1) $$J_{S}=\hat{n}\times \left(H_{S}-H_{I}\right)=\hat{n}\times H_{S},$$

(2) $$M_{S}=-\hat{n}\times \left(E_{S}-E_{I}\right)=-\hat{n}\times E_{S}.$$

The magnetic potential vector is related to the equivalent electric currents as

(3) $$A=\frac{\mu }{4\pi }{∯}_{S}^{\;}J_{S}(r^{\prime })\frac{e^{-jkR}}{R}dS\prime ,$$

while the electric potential vector is given in terms of the equivalent magnetic current

(4) $$F=\frac{\varepsilon }{4\pi }{∯}_{S}^{\;}M_{S}(r^{\prime })\frac{e^{-jkR}}{R}dS\prime ,$$

where R is the distance from point $r^{\prime }$ on the integral surface to the far-field observation point.

Based on the equations representing the relationships between electric, magnetic fields and potentials, and approximations in the far-field region [20], the total electric far-field E and the total magnetic far-field H can be written as

(5) $$E=E_{A}+E_{F}$$

(6) $$H=H_{A}+H_{F},$$

where the subscripts A and F indicate the component fields due to the A and F potentials, respectively. These component fields are expressed by

(7) $$E_{A}\approx -j\omega A,\;\;E_{F}\approx j\omega \eta \hat{r}\times F$$

(8) $$H_{A}\approx j\frac{\omega }{\eta }\hat{r}\times A,\;\;H_{F}\approx -j\omega F.$$

Partitioning the simulated volume into sub-volumes allows the calculation of the far-field response on a surface that covers each corresponding sub-volume. By selecting a sub-volume to exclusively cover the analyzed structural object, the far-field characteristic calculated based on its surface can represent the partitioned response of the object.

**Figure 7:**
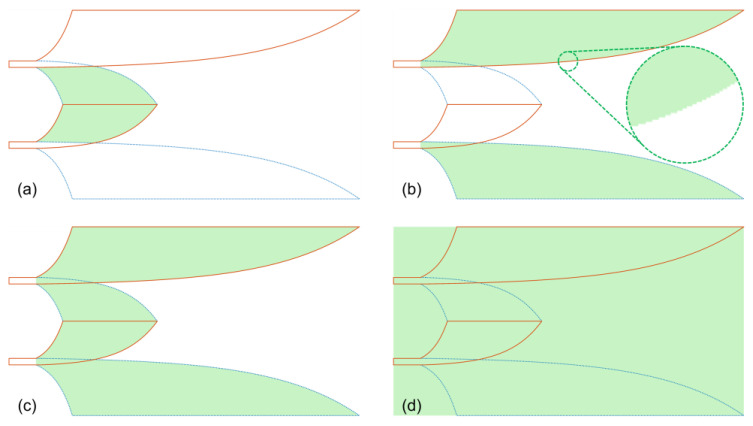
Structural sub-volumes for near-field to far-field transformation: (**a**) core section, (**b**) wings section, (**c**) wings & core and (**d**) whole structure.

In this study, specific sub-volumes of the reference antenna were chosen, comprising the core section, the lateral wings section, the combination of core and wings sections and the entire structure, as illustrated in the Figure 7. Additionally, the boundaries of these sub-volumes were also defined based on subsections of the wings, as detailed in Section 5. The boundary of each section was selected to encompass and enclose the two copper planar layers of the structure.

**Figure 8:**
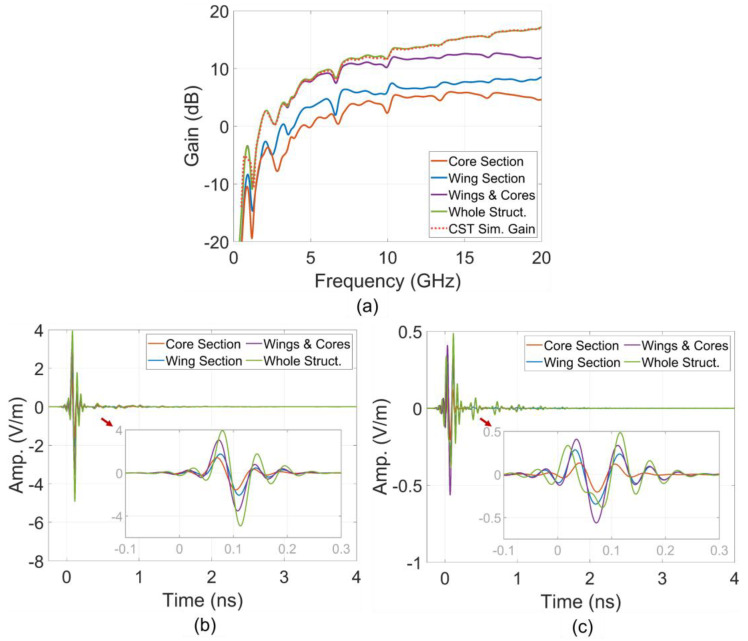
Structural partition (**a**) gains, (**b**) E-phi and (**c**) E-theta far-field impulse responses.

The electric field at a point located 10 m from the antenna origin in the main radiating direction was derived from the near-field response using an impulse bandwidth ranging from 0 to 20 GHz. This transformation was conducted across different structural sections. Additionally, realized gain was characterized for these structural sections within the same frequency band. The characteristics of gains in the frequency domain and electric far-field impulse responses in the time domain are shown in Figure 8. Moreover, the simulated gain of the entire simulation space, as characterized by CST, was included in the figure to validate the calculated characteristics. This validation was achieved by comparing CST gain with the gain calculated in the case of the entire structure.

The contribution of each structural partition to the antenna gain in the main radiating direction and the far-field response at a point in this direction is expressed by these characteristics. The contribution to energy transmission can be evaluated through derivatives such as average gain and total energy of the time response. ToAs of far-field propagation can be also determined from the time responses. For convenience in comparison with the time of the impulse at the excitation ports, the propagation time in free space corresponding to the 10 m distance from the antenna origin to the far-field sampling point was subtracted from the far-field time response.

### 2.4. Conventional Overall Characteristics

Typical overall antenna characteristics include S11, gain and far-field vectors. These characteristics provide insights into the overall responses of the entire analyzed structure. S11 represents the reflections from all regions within the structure back to the source. However, S11 only provides magnitude and phase information, and lacks spatial information, making it insufficient for analyzing propagation in 2D and 3D practical structures. Gain and far-field vectors of an antenna are the comprehensive far-field characteristics that describe the magnitude, intensity, phase and vector direction of the entire antenna’s radiation field in various directions. These characteristics are derived from near-field data through simulation. To conduct near-field analyses from a set of far-field vectors data in different directions, an inverse transform from far-field to near-field is required. However, losses of precisely spatial information in the transformation; and effects of obstacles and/or predominance of higher-order scattering in the analyzed structures significantly impact accuracy and reliability of this solution.

## 3. Analysis of Near-Field Characteristics on Structure With Strip Line Offset

**Figure 9:**
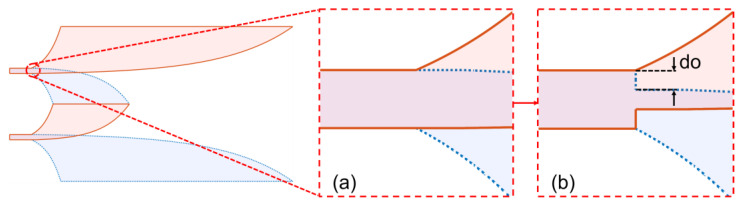
Zoom in structure (**a**) without and (**b**) with strip line offset.

The first improvement on the reference structure was introduced within the excitation strip line, where the EM field reaches its peak intensity and concentration. The propagation is directed along the flux of the EM flows originating from the waveguide excitation ports and is guided by the strip line. Particularly at the start of the Vivaldi structure, as illustrated in the magnified Figure 9a, there is asymmetric expansion and shrinking of the width between the top and bottom wings relative to the width of the parallel strips. In the reference structure, along the main Vivaldi curve, only a small width shrinking occurs at the first segment of the main Vivaldi edge. To amplify this effect, an offset do in wing width was introduced immediately after the strip, as shown in Figure 9b [21], to achieve a more substantial narrowing. This modification significantly changes the near-field EM flows and their responses in the far-field region. The outcomes of the near-field wavefront analyses conduct on the structure, and their structural effects on the far-field region, are presented in this section.

### 3.1. Near-Field Intensity and Wavefront Analysis

**Figure 10:**
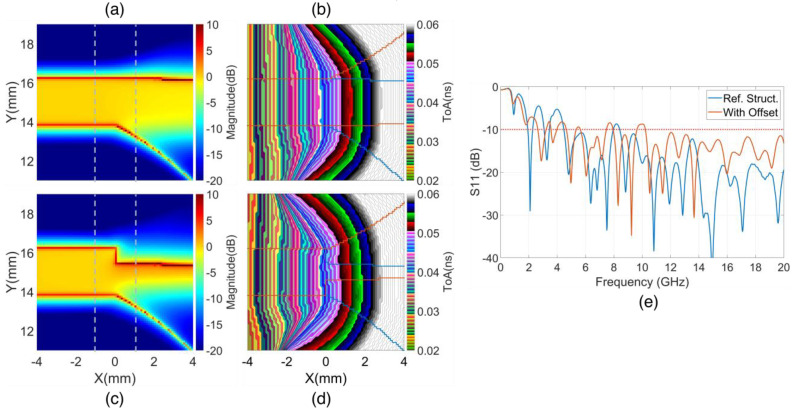
Zoomed-in image of Poynting magnitudes and ToAs of the first clusters on the structures (**a**,**b**) without, (**c**,**d**) with strip line offset and (**e**) S11 characteristics.

The magnitude of the first clusters and their ToA characteristics of Poynting vectors on the copper bottom layer inside the substrate of the reference structures are depicted in Figure 10a,b. These figures illustrate the concentration of parallel EM flows in the +x direction within the region guided by the strip. The expansion of the copper wing in the −y direction reorients a portion of EM flows spreading laterally to the wing. The small narrowing of the edge at X>2 mm, Y≈16 mm is also evidenced by an increase in the magnitude along the edge. 

The magnitude and ToAs on the structure with the strip line offset do are characterized in Figure 10c,d. These figures demonstrate that the reduction in the width at the offset reoriented EM flows from the strip line into higher density EM flows along the Vivaldi edges of the radiating slot. Despite the abrupt transition at the offset, the reflection back to the source is insignificant, as shown in the S11 characteristic in Figure 10e.

**Figure 11:**
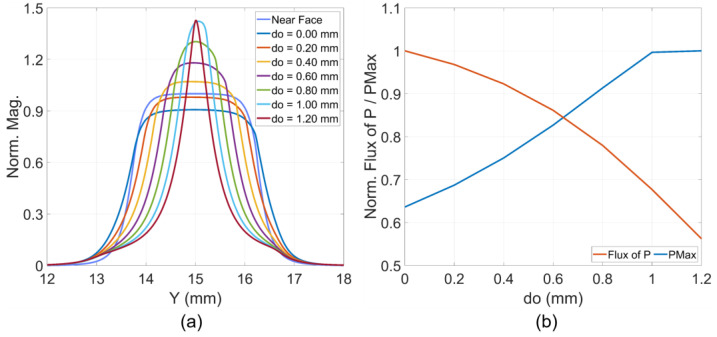
(**a**) Plot of normalized Poynting magnitudes on the cut lines X=±1 mm and (**b**) normalized maximum magnitude and flux of Poynting vectors.

To compare EM fluxes ahead and beyond the offset position, the characteristics at the faces with X=±1 mm were examined. The face nearer to the source, at X=−1 mm, lies within the strip line regions, while the face at X=1 mm is beyond the starting of Vivaldi curves or the offset position and is within the wing region. Figure 11a presents the magnitudes of Poynting vectors at the face X=1 mm and on the middle substrate layer, normalized by the maximum magnitude at the nearer source face at X=−1 mm, with strip line offsets do ranging from 0 to 1.2 mm. Figure 11b characterizes the normalized values of the maximum magnitude of Poynting vectors and the normalized flux values of Poynting vectors though the face X=1 mm over the same offset range as in Figure 11a.

The characteristics in Figure 11a simplify the features observed in Figure 10a,c. The results, parameterized by the do offset, demonstrate that increasing do leads to a concentration of EM energy density in the Vivaldi edge region, as clarified by the PMax characteristic in Figure 11b. Although there is an enhancement in the magnitude of the Poynting vectors at the edge, the flux of the Poynting vectors through the face at X=1 mm decreases with an increase in the do offset, as shown in Figure 11b.

These observations reveal two opposing trends. The first trend facilitates radiation at the Vivaldi edges due to increased intensity in this region. The second trend resists radiation because of the reduction in the area within the guiding or intersection part of the strips, which is necessary for transmitting EM energy to the radiating wings. These two trends suggest that an optimal do offset value likely falls in the middle of the analyzed do range 0–1.2 mm. This prediction is demonstrated in the subsequent analysis based on the near-field to far-field characteristics for gain and impulse responses.

### 3.2. Near-Field Effects to Far-Field Analysis

**Figure 12:**
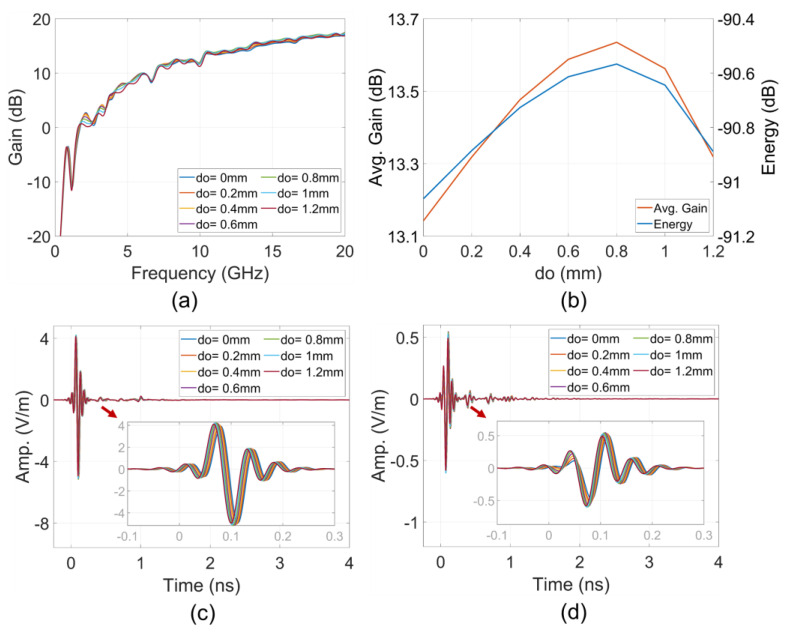
(**a**) Total gains, (**b**) far-field average gain, time response energy, (**c**) E-phi and (**d**) E-theta time response.

Analysis based on the near-field to far-field transform for the entire structure was conducted. The results, shown in Figure 12, indicate that the do offset significantly influences the gain and impulse responses intensity in the far-field. Figure 12b demonstrates that the average gain and energy of the far-field impulse responses, corresponding to different do offsets, suggest that do=0.8 mm is the optimal value for maximizing the average gain and time response energy in the far-field.

The ToA characteristics in Figure 10b,d show that the offset reorients the dominant EM flow from the edge of the strip to the Vivaldi edge, as depicted in Figure 10d. The ToAs of the clusters on the Vivaldi edge of the structure with the offset are a bit later than those on the reference structure. However, an opposite trend is observed in the ToAs of far-field time response, as shown in Figure 12c,d. With an increase in the do offset, the ToAs of far-field responses become earlier. This indicates that the do offset facilitates the EM flow propagating along the Vivaldi edges, enabling EM radiation to free space sooner than in the case without the offset. The faster propagation velocity in free space compared to the propagation velocity in the strip substrate contributes to the earlier ToAs at the far-field sampling point in the +x direction. This effect can be recognized by comparing the structures in Figure 9a,b. With the do offset, the area for guiding the EM field in the structure, similar to the strip line ahead the offset position, is reduced, and the radiating area of the Vivaldi edge is moved closer to the offset position. While the total distance from source plane to the far-field sampling point remains unchanged, the increased distance of propagation in free space leads to earlier ToAs. Additionally, the extension of the radiating length proportion of the Vivaldi edge caused by the do offset also contributes to the increased gain of the improved structure.

## 4. Analysis of Core Length Effects to Near-Field Characteristics

**Figure 13:**
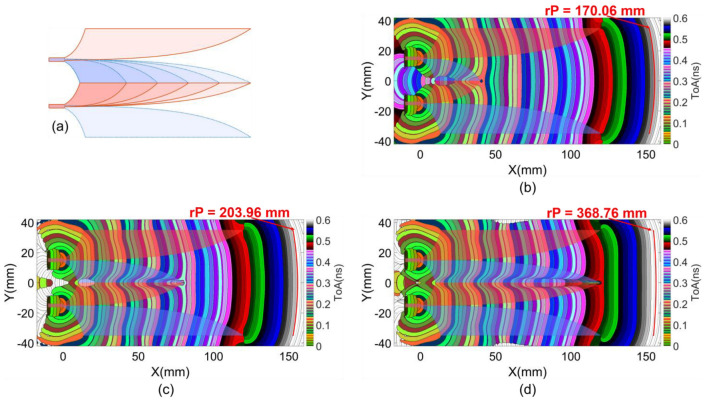
(**a**) Structure with different core lengths and first cluster ToAs with (**b**) 40 mm, (**c**) 80 mm, and (**d**) 120 mm core lengths.

In this section, based on the optimized structure of the previous section, the analyses on the effects of the length geometry feature of the core formed by the two center wings [14] on the antenna responses in near-field and far-field were implemented. Figure 13a illustrates the core length parameterization over a range of 40 to 120 mm with 20 mm step increments.

### 4.1. Near-Field Wavefront Analysis

The ToA characteristics of the first clusters of Poynting vectors analyzed on the middle layer of the substrate are illustrated in Figure 13b–d, corresponding to core lengths of 40, 80 and 120 mm, respectively. In regions along segments from the offset position to the middle of the Vivaldi edges, EM flows from the source propagate along and are guided or constrained by the metal Vivaldi edge strip of the wings. These source flows also generate first-order scattering EM flows along these metal strips. In these regions, the source flows and the first-order scattering flows are more dominant than higher-order scattering flows. These first clusters are not significantly affected by other first-order scattering flows, such as reflecting flows from the end of the Vivaldi wing.

Additionally, with the extension of the Vivaldi curves in the ±y directions, the length of the propagation path along the metal edge strip is longer than that in the +x direction in the slot. Consequently, the ToAs of the constrained flows along the metal strip are later than those of flows scattered at lower x coordinates and propagating in the +x direction within the dielectric of the slot. This is expressed in the curvature of the wavefronts at the Vivaldi slots. The balanced excitation of the two Vivaldi slots facilitates the serial connection of two parts of the wavefronts from the lower and upper Vivaldi slots, propagating into the common radiating aperture.

Lengthening the core not only enhances geometric balance for the two slots but also extends the propagation path length of the constrained flows on the core wing strips, facilitating more first-order scattering flows from these parts of the structure. These effects are evident in the enhanced flatness and the later ToAs at the radiating aperture as the core length increases, as shown in Figure 13b–d. The curvature of the wavefronts was approximated by a fitted circle at the radiating antenna aperture. The trend of wavefront flatness enhancement over all analyzed core lengths is presented in Table 1.

### 4.2. Partitional Near-Field Effects to Far-Field Analysis

In this section, the core section as described in Figure 7a of the structures, with a core length ranging from 40 to 120 mm, was analyzed as the sub-volume for near-field to far-field transformation. This analysis examined the effects of this part on the far-field region at the point 10 m away from the origin in the +x direction.

**Figure 14:**
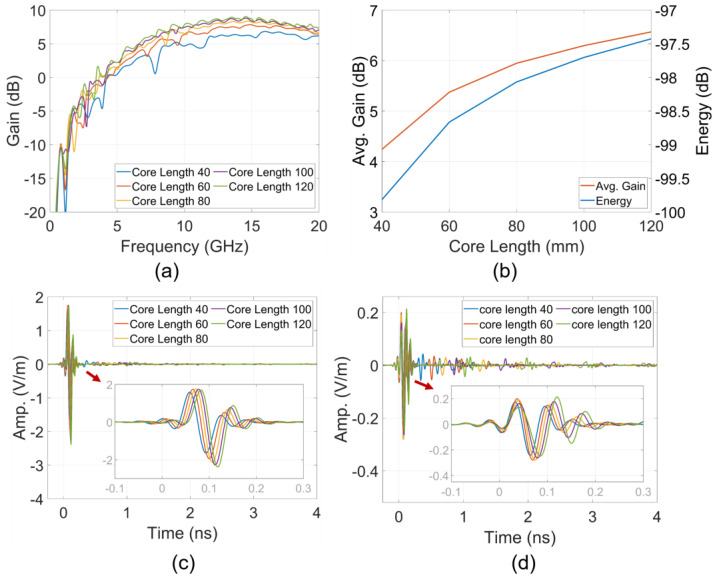
(**a**) Partitional gains, (**b**) average partitional gains and time impulse response energy, (**c**) E-phi and (**d**) E-theta.

The result in Figure 14a shows the increasing trend in core partition gains across almost the entire frequency band as the core length increases. This trend is further clarified by the results shown in Figure 14b, which clearly indicate that the average gains and energies of far-field time responses increase with the elongation of the core. The effectiveness of increasing the core length from 40 mm to 120 mm results in an approximate 3 dB improvement in the partitional average gain and response energy.

The detailed results in Figure 14c,d for the core partition far-field responses reveal not only an increase in amplitude with longer core lengths but also an increase in ToAs. These findings validate the near-field wavefront analyses, emphasizing the contributions of the core, particularly the core edge strips. The core edge strips facilitate first-order scattering flows or radiating flows and also extend the ToAs of the wavefronts, as discussed in the previous section.

## 5. Analysis of Near-Field Characteristics on Structure With Lateral Comb Slots

**Figure 15:**
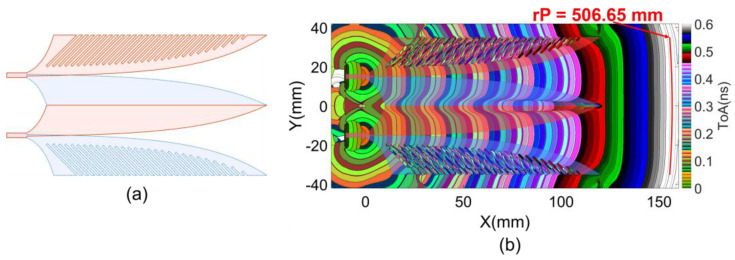
(**a**) Structure with lateral comb-slots and (**b**) first cluster ToAs of Poynting vectors.

In continuation of the optimization process outlined in the previous two sections, another improvement was added to the existing structure. This enhancement involves an addition of 29 parallel slots etched into each of the two lateral copper wings at an angle of 45 or −45 degree in the +x direction [14]. The lengths of these slots were adapted to the Vivaldi curve to maintain a consistent 4 mm width strip along the Vivaldi edge of the lateral wing as illustrated in Figure 15a. The impacts on near-field and far-field regions of these lateral comb slots are analyzed in this section.

### 5.1. Near-Field Wavefront Analysis

The ToAs wavefront result in Figure 15b indicates that, within the regions of the Vivaldi edge strip and dielectric slot, the wavefronts produced by the source EM flow and the first-order scattering EM flows are quite similar to those in the previous structure shown in Figure 13d. However, the lateral comb slots introduce complex scattering EM flows within their regions. This complexity also affects the curvature of the wavefront part stretching across the boundary line between the Vivaldi main strip and the Vivaldi slot, specifically increasing the curvature.

The increase in the wavefront curvature evidences an increase in density of scattering flows in the Vivaldi slot, resulting in earlier ToAs compared to the constrained flow on the Vivaldi strip, which exhibits later ToAs. The scattering in the comb slots regions contributes to this increased density of scattering flows in the Vivaldi slot. This results in a significant improvement of wavefront flatness at the radiating aperture, with a wavefront radius reaching up to 506.65 mm.

Additionally, parallel lines formed by the comb slots with varying lengths facilitate higher-order scattering or resonance on individual lines and/or groups of lines. This extends the response time and also improves antenna performances at low frequencies. These impacts of the comb slots on response improvements of the antenna are more clearly observed in the partitional far-field analysis.

### 5.2. Partitional Near-Field Effects to Far-Field Analysis

**Figure 16:**
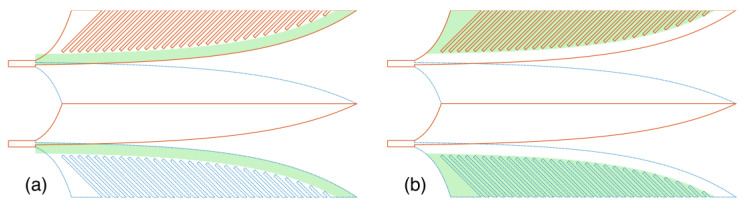
Structural sub-volumes for near-field to far-field transformation: (**a**) main Vivaldi strip section and (**b**) remaining or comb slots section.

Volumes containing the main Vivaldi strip with a 4 mm width and the comb slots in the lateral wings were selected as sub-volumes for analysis of the antenna with the comb slots, as illustrated in Figure 16. To facilitate a comparison with the antenna without the comb slots, the two lateral wings without the comb slots were divided into the same two sections: the main Vivaldi strip section and the remaining section of the lateral wings.

**Figure 17:**
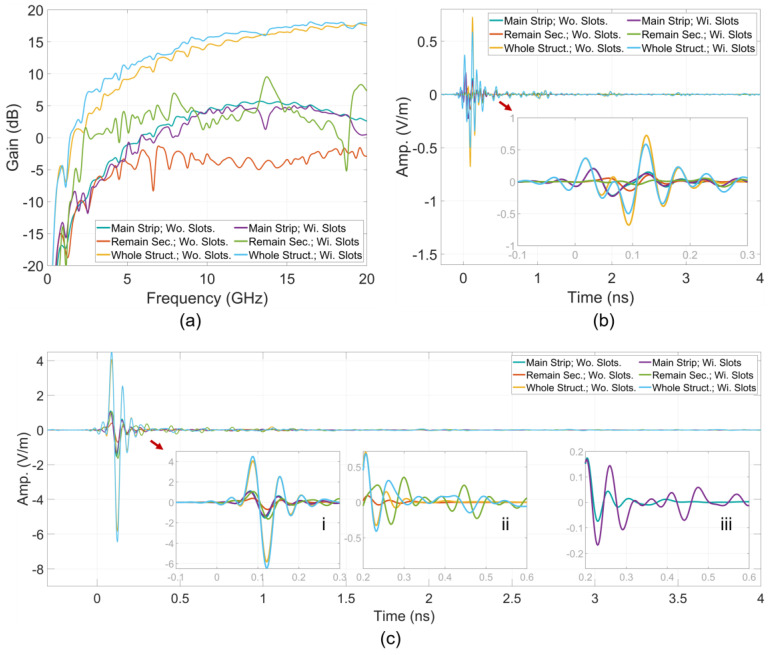
(**a**) Partitional and total gains, (**b**) E-theta and (**c**) E-phi time impulse responses.

The results of partitional gains for these sections, shown in Figure 17a, indicate that the 4 mm main strip sections of both antennas—those with and without the comb slots—contribute similarly to the gains feature across the entire frequency band. However, the remaining section of the wings with the comb slots contributes significantly higher partitional gains compared to the same section of the antenna without the comb slots. The gains contributed by the partition comb slots are also high at lower frequencies such as around 2 GHz. This supports the earlier discussion about the impact of the comb slots on scattering flows.

The gain of the entire structure further highlights the contribution of the comb slots to improving the antenna’s performance. The amplitudes of the far-field impulse responses, shown in Figure 17b,c, also present as the feature in the partitional gains. Analysis of the lengthening in response time of these signals, as depicted in Figure 17c(i–iii), demonstrates that the comb slots facilitate higher-order scattering or resonance within the lines of these regions, resulting in a longer response time for these signals.

**Figure 18:**
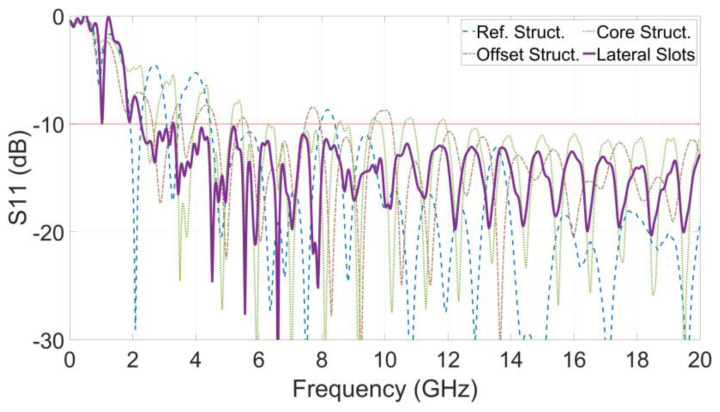
S11 Characteristic on structures.

During the research Section 3, Section 4 and Section 5, the near-field wavefront, far-field response and gain characteristics of the antennas were improved step by step. For a complete presentation of near-field response features of the antennas, the S11 characteristic was evaluated on the reference structure and the improved structures of each step as in Figure 18. This shows that the offset in the excitation line does not cause a significant adverse effect on S11, and the increase in core length and adding the lateral comb slots on the structure significantly enhance S11 performance, especially in the lower frequency band.

## 6. Conclusions

The near-field characteristics of intensity, ToA of EM cluster, partitional gain in frequency domain, partitional far-field impulse response in time domain and their derivatives are proposed. The approach allows the interpretation of the EM propagation process within the structure as features such as the dominant regions, dominant flows, ToAs/velocities/wavefronts of the flows and far-field impulse responses of the localized structural regions. This consequentially allows a simplification of the propagation process into a simpler and more manageable model, which elucidates the role and impact of the structural geometries or the structural details on the EM response features or the near-field characteristics. The detail or locality in spatial distribution of the characteristics facilitates the analysis of the effects of sub-structures of the geometry. The geometry, correlation and extrema feature of the near-field and partition characteristics along with their derivatives are used as criteria for optimizing structural parameters during the design process. The optimization of these parameters and the effectiveness of the improved structures are demonstrated using both these characteristics and the conventional overall characteristics.

The analyses based on near-field and partitional near-field to far-field characteristics simplifies near-field propagation models, thereby enhancing the understanding of near-field propagation processes in the double-slot antipodal Vivaldi antennas and contributing to their improvement. This methodology is also promising for exploring EM propagation processes in various other traveling wave antennas.

## Figures and Tables

**Table 1:** Radius of wavefronts over core lengths.

Core Length (mm)	Radius of Wavefront (mm)
40	170.05
45	170.34
60	173.91
80	202.96
100	245.23
120	368.76

## Data Availability

Data are contained within the article.
